# Point-of-care ultrasound in the head and neck region

**DOI:** 10.1007/s10396-022-01266-8

**Published:** 2022-10-26

**Authors:** Madoka Furukawa, Kaori Hashimoto, Yuka Kitani, Manatsu Yoshida

**Affiliations:** Department of Head and Neck Surgery, Kanagawa Cancer Center, Yokohama, Japan

**Keywords:** Head and neck, Point-of-care ultrasound, Neck tumor, Upper airway obstruction, Swallowing function

## Abstract

Point-of-care ultrasound (POCUS) in the head and neck region plays a particularly significant role in the diagnosis and treatment of upper airway stenosis, swelling, and painful diseases in the neck, and in the evaluation of swallowing function. Therefore, it should be performed in various medical settings beyond the boundaries of the clinical department such as general medicine, emergency medicine, anesthesiology, orthopedics, and pediatrics. The target diseases are salivary gland disease, lymph node disease, pharyngeal disease, laryngeal disease, esophageal disease, thyroid disease, and dysphagia and dyspnea due to various causes. Head and neck POCUS is an extremely useful diagnostic method for both patients and doctors, and its use is expected to become more widespread in the future.

## Introduction

Point-of-care ultrasound (POCUS) is an examination procedure for doctors to make an ultrasound diagnosis as part of the initial examination and determine a medical treatment plan in primary care. Head and neck anatomy is complicated as various organs with various functions are confined to a small region, and a variety of diseases and conditions can arise in this region. In the head and neck region, lesions may occur near the body surface and can be easily visualized by ultrasound. Thus, POCUS is effective in obtaining accurate information rapidly at the early stage of treatment.

POCUS in the head and neck region plays a particularly significant role in the diagnosis and treatment of upper airway stenosis, swelling, and painful diseases of the neck. Therefore, it should be incorporated into daily medical care beyond the boundaries of the field, including general medicine, emergency medicine, anesthesiology, orthopedics, and pediatrics, as well as medical care by specialized departments of the head and neck. However, a comprehensive POCUS education system and training curriculum have not yet been established [1–4].

## Ultrasound anatomy of the head and neck

The anatomy of the head and neck is complicated and difficult to understand. Therefore, it is necessary to have sufficient knowledge to correctly identify what is visualized on ultrasound. In addition, since there are individual differences in the thickness and length of the neck, it must be taken into consideration that the depth and size of organs seen on ultrasound images varies from patient to patient.

Patients with a severely swollen neck, having difficulty breathing, or severe pain may have restrictions on their posture and position. When the examination is performed with the patient in an inadequate position, it may not be possible to determine which part of the neck is currently visible. As a solution in such cases, it is recommended to first find the pulsating common carotid artery in the cervical transverse view that is easily recognizable in the neck. After detecting the common carotid artery, the thyroid gland and trachea and other structures can be identified by confirming the positional relationship.

## Equipment and procedures required for POCUS of the head and neck

### Ultrasound diagnostic device and probe

A linear probe with a frequency of around 10 MHz and a field of view of around 40 mm is suitable for head and neck POCUS. A lightweight and thin-shaped probe makes it easy to grip and adjust the angle, and it is easy to apply to various surfaces of the neck. Head and neck POCUS may sometimes need to be performed in a difficult position in various environments, so it is best to use a system that is easy to operate. Since POCUS must be performed in various environments and even in inconvenient situations, small machines with good operability (e.g., portable echo, pocket echo, cordless echo) are more suitable than large high-end models with multiple functions [5–8].

### Patient position and posture

Naturally extending the patient's neck in the supine position makes it easier for the probe to fit the skin of the neck and makes it easier to scan the entire neck. The supine position with the patient's chin slightly raised and both knees bent is specifically recommended because it can relax the anterior neck, chest, and abdomen, and it does not interfere with natural breathing movements. Shoulder pillows are not needed as they overextend the patient's neck and cause tension in the neck muscles. Of course, if the patient has painful symptoms, it is important to devise a comfortable posture according to the patient's condition. If the patient has difficulty in the supine position due to severe pain or severe dyspnea, the examination may be performed in the sitting or lateral decubitus position. In these cases, it is necessary to consider that there are some limitations in the observable region and that various organs are in a different positional relationship in the sitting position or the lateral decubitus position than in the supine position. In the sitting position, the mandible lowers due to the weight of the skull, which makes it exceedingly difficult to examine the submandibular region and the upper neck area. Also, the organs in the lower neck are pulled into the mediastinum. Furthermore, it should be noted that in the sitting position, the blood vessels of the venous system collapse, which makes it difficult to identify the blood vessels and their blood flow. In addition, when performing an examination in a sitting or lateral decubitus position, the doctor is also forced into an unnatural posture, which makes a lengthy examination difficult. The posture of the doctor performing the examination should also be as comfortable as possible.

### Scanning methods

**Fig. 1:**
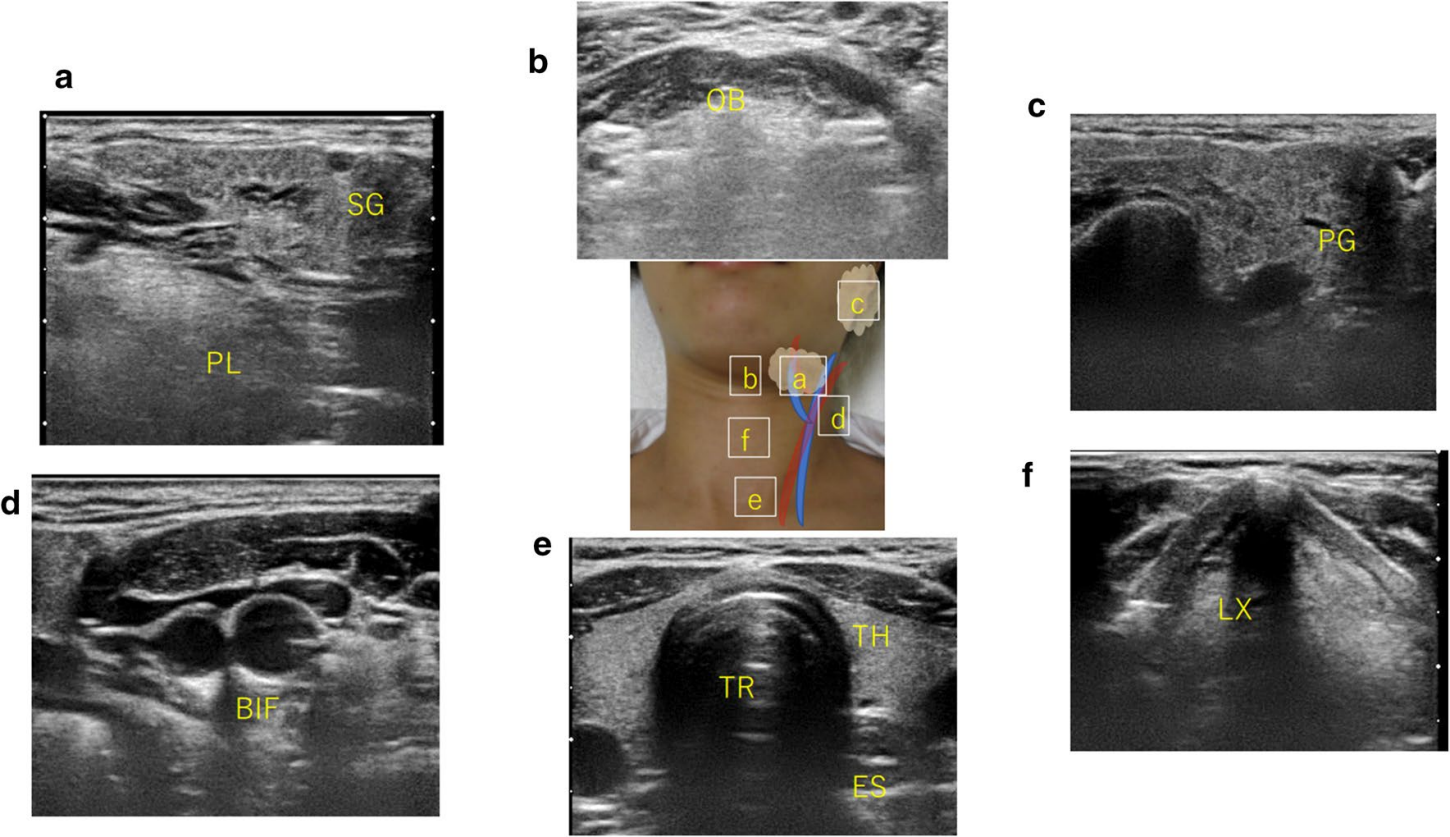
Basic image of the neck (systematic cervical ultrasonography). *SG* submandibular gland, *PL*: pharyngeal lateral wall, *OB*: oral base, *PG*: parotid gland, *BIF*: carotid bifurcation, *TR*: trachea, *TH*: thyroid gland, *ES*: esophagus, *LX*: larynx. The probe should be continuously scanned to ensure that it passes through these sites, and the left and right necks should always be observed. Observe in a manner that does not overlook changes in lymph nodes, blood vessels, and muscles along the way. **a** Submandibular region (submandibular gland, facial arteriovenous, oropharyngeal sidewall). **b** Submental region (oral floor muscles, base of tongue). **c** Parotid region (parotid gland, mandible, masseter muscle). **d** Carotid bifurcation (external carotid artery, internal carotid artery, internal jugular vein). **e** Anterior neck (thyroid, common carotid artery, internal jugular vein, cervical esophagus). f Larynx

When performing an ultrasound examination of the neck, a linear probe is usually used to continuously scan the left and right sides and the midline of the neck. The cervical organs are covered with fascia and are bounded by layers of each fascia, and various organs such as blood vessels, muscles, and pharynx and larynx run vertically to connect the head with the thorax and shoulders. Therefore, the probe is moved up and down while looking at the transverse-sectional image of the neck, and the location of the lesion is confirmed while following the continuity of the blood vessels and fascia. In addition, since the submandibular gland and the parotid gland exist at the end of the neck, it is necessary to consider the anatomical features when scanning. These series of procedures are called ‘‘systematic cervical ultrasonography’’ as a procedure for observing the entire neck briefly (Fig. 1). This is a method for observing the left and right sides and the midline neck, as well as the parotid gland and the submandibular gland in a continuous and concise manner, and it is a procedure that can yield various findings of the entire neck including accidental findings. Therefore, it is a procedure that should be performed routinely to cover the entire neck with head and neck POCUS.

In an emergency such as upper airway obstruction with severe dyspnea, there may only be time for an airway examination, but no matter how urgent, you should try to observe the entire neck as much as possible.

## Head and neck POCUS in various clinical situations

### Head and neck POCUS of patients complaining of dyspnea

One of the most urgent conditions in the head and neck is upper airway obstruction. Diseases that cause upper airway stenosis include acute upper respiratory tract inflammation, laryngeal tumors, pharyngeal tumors, bilateral vocal cord midline fixation due to bilateral vocal cord paralysis, and foreign bodies in the larynx and trachea. Head and neck POCUS is often useful in diagnosing upper airway obstruction when specialized laryngeal fiberscope examination is not available. Not only abnormal findings of the pharynx, larynx (vocal cords, epiglottis, subglottic), and trachea but also submucosal lesions and abnormalities can be easily detected [9–11]. POCUS of the upper respiratory tract is useful in anesthesiology and emergency outpatient departments, but it is necessary to correctly understand the upper airway structures while properly linking the POCUS findings to the fiberscope findings including the pharyngeal and laryngeal movements and functions.

When patients complain of dyspnea, diseases that occur in the lower respiratory tract such as the bronchi and lungs are usually considered, but upper airway POCUS should also be performed as primary care for dyspnea. Swelling of the laryngeal mucosa; tumors of the larynx, trachea, and its surroundings; thyroid lesions; deformity of the thyroid and cricoid cartilage; and vocal cord paralysis should be checked as pathological conditions. If an abnormality in the upper airway is suspected based on head and neck POCUS, professional treatment by an otolaryngology head and neck surgeon should be arranged immediately. Among the diseases that cause upper airway obstruction, in the case of acute epiglottitis and laryngeal cancer, there is a risk of airway edema and bleeding due to inadvertent intratracheal infusion, which promotes airway obstruction and leads to choking.

#### POCUS of the larynx

**Fig. 2:**
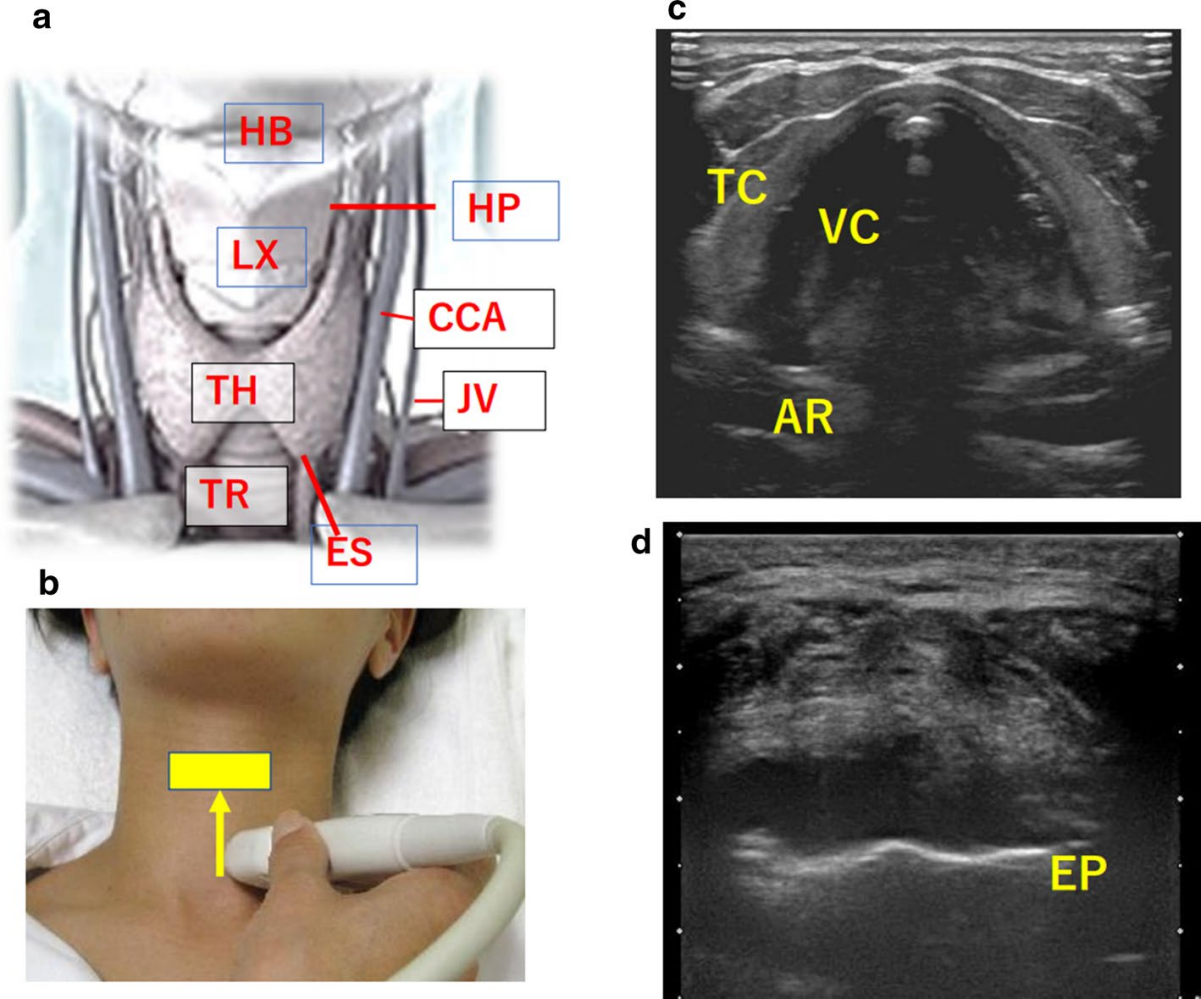
POCUS of the anterior neck *HB* hyoid bone, *LX* larynx, *TH* thyroid gland, *TR* trachea, *HP* hypopharynx, *ES* esophagus, *CCA* common carotid artery, *JV* internal jugular vein, *VC* vocal cord, *TC* thyroid cartilage, *AR* arytenoid cartilage, *EP* epiglottis cartilage. **a** Anterior neck anatomy. **b** Anterior neck probe operation. **c** Transverse view of the anterior neck: Ultrasound image of the larynx (vocal cord level). **d** Transverse view of the anterior neck: Ultrasound image of the larynx (supraglottic level)

In POCUS of the larynx, movement of the vocal cords, properties of the vocal cords and surrounding tissues, and findings of the epiglottis are observed (Fig. 2). The thyroid cartilage, which forms the framework of the larynx, is soft and moist in young people and females of all ages, and has little anterior protrusion, so mucosal lesions in the laryngeal cavity and arytenoid cartilage movement can be easily detected through the thyroid cartilage. On the other hand, since cartilage ossification is pronounced in elderly men, it is difficult to observe from the front of the neck. In that case, it is recommended to observe the left and right vocal cords separately through the thyroid plate.

#### POCUS for acute epiglottitis

**Fig. 3:**
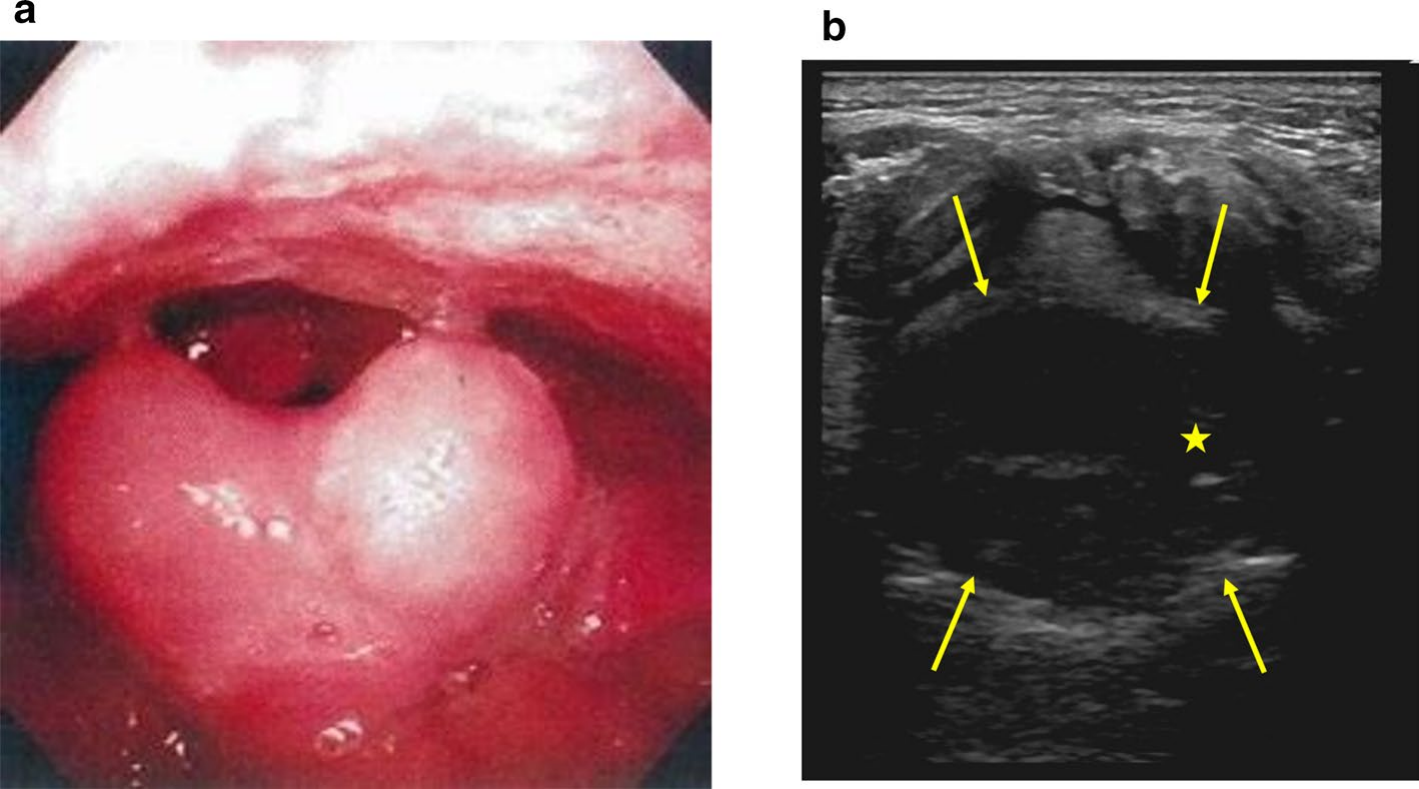
Acute epiglottitis It is caused by bacterial or viral infections. It is a disease that requires emergency medical care such as securing an emergency airway because the symptoms worsen rapidly. **a** Laryngeal fiberscope findings: The epiglottis is swollen **b**. Transverse view of the anterior neck: Swelling of the mucosa (↑) surrounding the epiglottis cartilage block filled star is observed

The epiglottis is observed in a transverse section view between the thyroid cartilage and the hyoid bone or from the upper edge of the hyoid bone between the base of the tongue and the larynx. In a normal epiglottis, the flat epiglottis cartilage appears as a hyperechoic line, but in patients with acute epiglottitis, the epiglottis becomes edematous with inflammation following infection-induced pharyngitis and the mucosa surrounding the epiglottis swells and thickens. appearing as a hypoechoic sphere obstructing the airway on POCUS (Fig. 3).

#### POCUS for laryngeal cancer

Laryngeal cancer also causes airway obstruction. In the type that progresses submucosally, laryngeal cancer invades the thyroid cartilage slowly without symptoms such as hoarseness or pain, and airway stenosis may gradually progress unnoticed. It is also important to know that laryngeal cancer is a common cancer among smokers and is often misdiagnosed as asthma or chronic obstructive pulmonary disease. Since dyspnea and wheezing during inspiration are findings peculiar to upper airway obstruction, POCUS of the larynx should be performed to check for neoplastic lesions and thyroid cartilage invasion. Laryngeal cancer that has invaded the thyroid cartilage can be very easily seen with ultrasound.

#### POCUS for vocal cord paralysis

The main causes of vocal cord paralysis are surgical injury and malignant tumor infiltration to the recurrent laryngeal nerve. When the vocal cords on both sides are fixed in the median position, upper airway obstruction occurs, and dyspnea appears. To diagnose vocal cord paralysis with POCUS, it is necessary to carefully observe and compare the movements of the vocal cords and arytenoid cartilage on both sides.

#### POCUS in the subglottic region and trachea

Since stenosis and obstruction from the subglottic region to the trachea cannot be seen on laryngeal fiberscope examination, POCUS should be used to confirm the presence or absence of airway stenosis. In this region, the thyroid cartilage, the cricoid cartilage, and the tracheal cartilage are continuously observed in a transverse section view to diagnose the presence or absence of lesions in the cartilage lumen. This is a useful technique for diagnosing subglottic neoplastic lesions, foreign bodies, cricoid cartilage, and tracheal cartilage fractures and deformities.

### POCUS for neck swelling and neck pain

Head and neck POCUS plays a significant role in the primary care of patients who complain of cervical swelling, neck mass, and neck pain.

Since it is difficult to communicate symptoms in the neck, patients often cannot correctly convey symptoms such as tumors, swelling, and pain, or the site with symptoms, and the chief complaint is often vague and ambiguous. The main complaints that are often expressed are hoarseness, difficulty speaking, difficulty breathing, shortness of breath, feeling pain, swollen neck, lump in the neck, pain in the neck, pain in the throat, pain on swallowing, discomfort in the throat, difficulty swallowing, and easy aspiration. Sometimes, the site where the patient complains of symptoms and the site where the lesion occurs may be different. Observing the entire neck with POCUS, keeping in mind all potential diseases and their characteristics, is a shortcut to a correct diagnosis.

Visual inspection and palpation are usually performed on the neck, but palpation may be difficult depending on the condition of the patient's skin and muscles and the depth of the lesion. In such cases, POCUS can play a significant role, but if there are open wounds, subcutaneous emphysema, and/or hematoma at the examination site, it may be difficult to diagnose even with POCUS. In such cases, the need for examination by another modality such as CT must be properly decided.

Diseases that cause cervical swelling and pain include inflammation and tumors of the cervical lymph nodes, submandibular glands, parotid glands, thyroid gland, pharynx, larynx, and cervical esophagus. It should be noted that patients with thyroid disorders, especially subacute thyroiditis, often complain of a sore throat and pain on swallowing rather than pain in the thyroid area. In addition, blood vessels such as the common carotid artery and internal jugular vein, muscles and nerves also cause neck swelling and pain.

#### POCUS around the submandibular and parotid glands

Salivary gland diseases such as sialolithiasis, acute and chronic inflammation, benign and malignant salivary gland tumors, and salivary gland autoimmune diseases cause neck swelling and neck pain. Since malignant tumors account for about 30% of salivary gland tumors, and it is also known that they change from benign to malignant, appropriate management is required [12]. If a salivary gland tumor is suspected based on POCUS, a professional consultation should be arranged. In addition, if POCUS indicates IgG4-related disease or Sjogren's syndrome, which are autoimmune salivary gland diseases, systemic symptoms should be pursued.

#### POCUS for cervical lymphadenopathy

Lymph nodes are widely distributed in the neck and swell due to various causes such as reactive lymphadenopathy due to inflammation and infection, metastasis of cancer, and malignant lymphoma [13]. In head and neck POCUS, it must first be determined whether the detected mass is a lymph node. When distinguishing whether a mass is a lymph node or not, attention should be paid to the presence of an echogenic hilum (fatty hilum) near the lymph node hilum as the structure that is most easily visualized on ultrasound in the lymph node. This structure is an image of the complex structure of arteries and veins, efferent lymph vessels, and surrounding connective tissue and adipose tissue. If this structure is found in a part of the mass, the mass can be judged to be a lymph node. In the case of reactive lymph node swelling, the lymph node structure remains normal, and in malignant lymphoma, the lymph node hilar structure develops more than normal, and the blood vessels inside the lymph node become thicker and blood flow increases. In the case of lymph node metastasis of cancer, normal lymph node areas, including the lymph node structure, are either driven to the inner edge of the lymph node or surrounded by cancer metastatic lesions. Since cervical lymphadenopathy in adults is likely to be a malignant disease, when lymphadenopathy with an abnormal lymph node structure is found during head and neck POCUS, referral to a specialist is recommended.

#### POCUS of thyroid gland and surrounding area

**Fig. 4:**
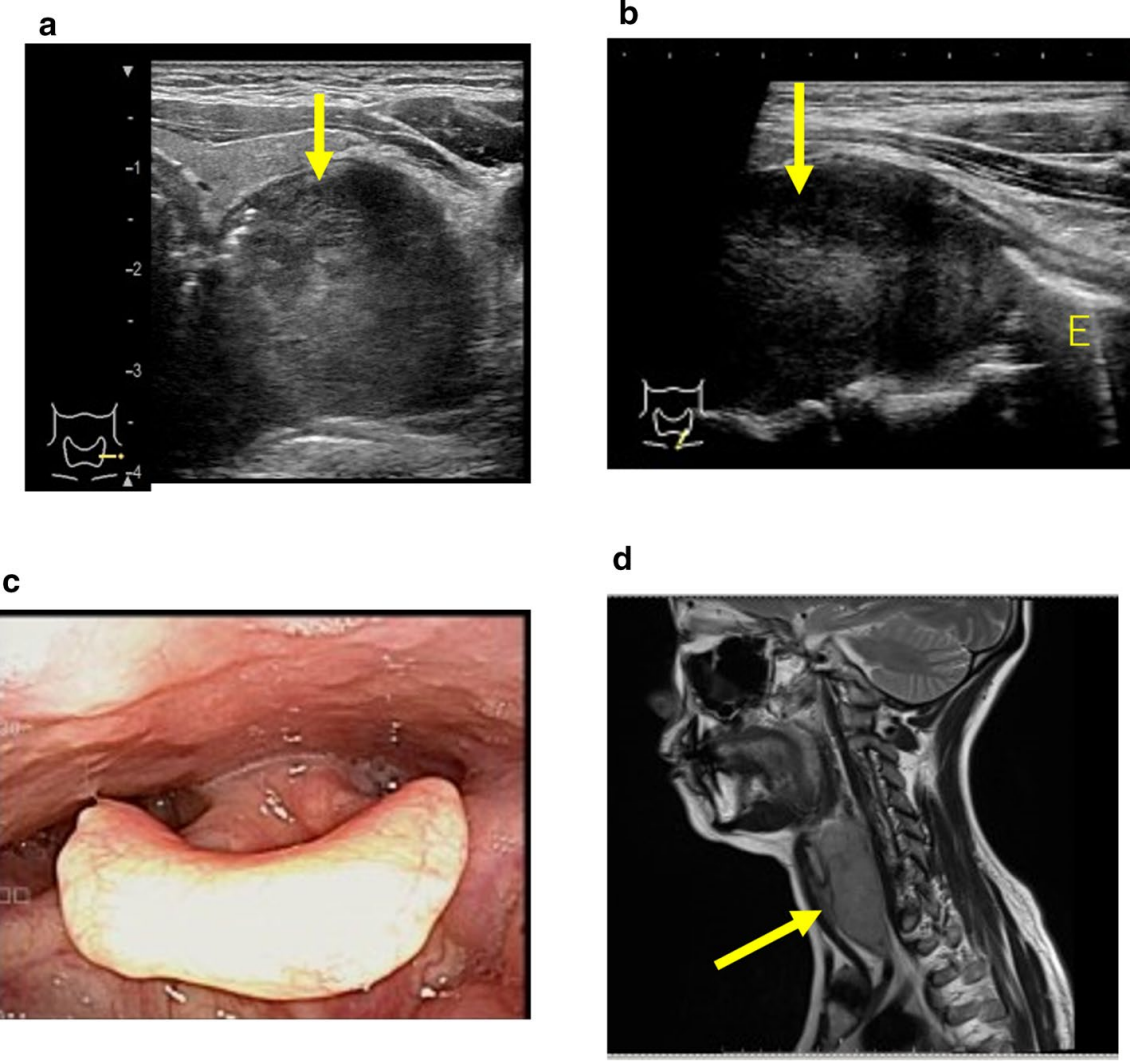
Advanced cancer of the cervical esophagus. **a** Transverse view of the lower part of the anterior neck: A tumor (↑) was found on the dorsal side of the left lobe of the thyroid gland. **b** Sagittal image of the lower part of the anterior neck: The tumor (↑) led to the cervical esophagus (E). **c** Laryngeal fiberscope findings: No obvious neoplastic lesions can be pointed out. **d** Cervical MRI findings (sagittal section): A tumor (↑) was found from the posterior hypopharyngeal ring to the cervical esophagus

The thyroid gland, which is located in the anterior neck in front of the trachea and larynx, can be easily observed by applying a probe to the anterior neck. Benign lesions that are not the target of aggressive treatment are often visible, so proper judgment is required. By performing POCUS, diagnosis of diffuse thyroid disease such as chronic thyroiditis (Hashimoto's disease) and Basedow's disease (Graves’ disease), benign thyroid tumors, thyroid cancer, parathyroid disease, cricoid retropharyngeal cancer, and cervical esophageal cancer is possible (Fig. 4).

### POCUS in swallowing function evaluation

**Fig. 5:**
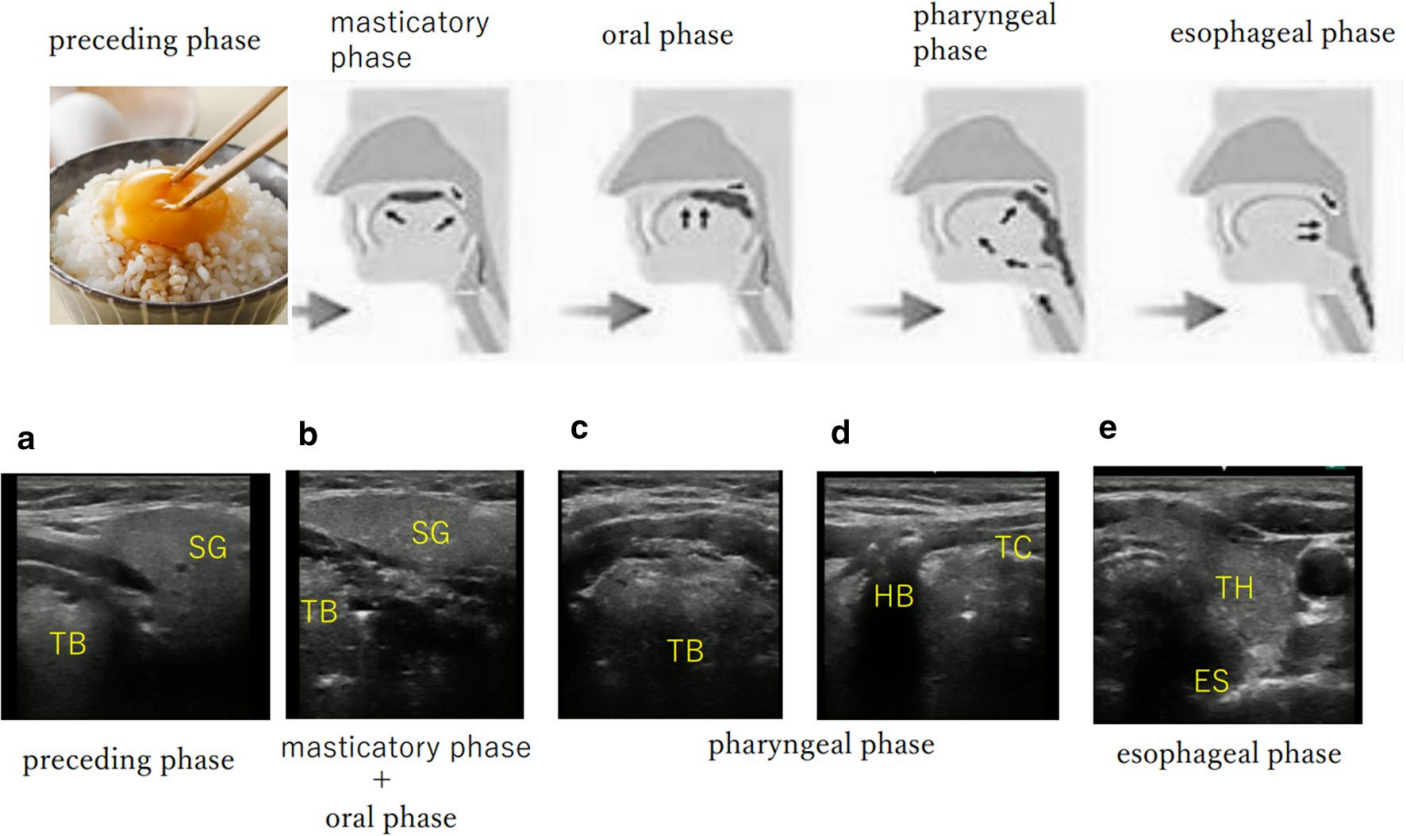
Evaluation of swallowing function by POCUS of the neck *SG* submandibular gland, *TB* base of tongue, *HB* hyoid bone, *TC* thyroid cartilage, *TH* thyroid gland, *ES* esophagus. **a** Image from the submandibular region: Salivation is promoted in the preceding phase. **b** Image from the submandibular region: A large amount of saliva is observed to be released from the submandibular gland, and the movement of the tongue mixes food and saliva. **c** Image from the submental region (transverse). **d** Image from the submental region (sagittal). **e** Transverse view of the lower part of the anterior neck

It is also possible to evaluate swallowing function with POCUS (Fig. 5). By observing the movement of the lateral wall of the oropharynx, the base of the tongue, and the cervical esophagus with ultrasound, it is possible to easily evaluate swallowing function anytime and anywhere. Swallowing movement consists of a very subtle combination of reflexes that are performed unconsciously using the entire oropharyngeal function, and are divided into the preceding phase, the masticatory phase, the oral phase, the pharyngeal phase, and the esophageal phase. When eating, each movement is subtly combined and repeated.

From the preceding phase to the oral phase, observe the oral cavity from the submandibular area with POCUS. Visual information during the preceding phase stimulates salivation. During the masticatory phase, a large amount of saliva is observed to be released from the submandibular gland, and the movement of the tongue mixes food and saliva. During the oral phase, the base of the tongue rises and feeds the food mass into the pharynx. During the pharyngeal phase, a mass of food is sent from the pharynx to the esophagus. Observations from the submandibular area show elevation of the base of the tongue and contraction of the lateral wall of the pharynx. Observations of the submental cervical transverse section view show large movements of the base of the tongue and the collapse of the epiglottis. In addition, the anterior cervical longitudinal section view shows the elevation of the hyoid bone and thyroid cartilage toward the head. In the esophageal phase, you can see that the food mass sent from the pharyngeal phase vigorously passes through the cervical esophagus. By using ultrasound, it is possible to perform the examination with the contents of the meal that the patient usually eats in the environment where the patient usually lives, even if it is not a special examination room.

## Conclusion

In patients with dyspnea, head and neck POCUS should be used to diagnose the condition of upper airway obstruction and its causes. When performing POCUS in patients with neck swelling or neck pain, the entire neck should be examined as one organ. It is important to diagnose the presence or absence of lesions and the location of lesions, and to perform qualitative diagnosis based on comprehensive knowledge of head and neck anatomy and diseases. With the miniaturization and weight reduction of ultrasonic diagnostic equipment, the range of utilization of POCUS will be further expanded, such as swallowing function evaluation and its rehabilitation and home medical care. Head and neck POCUS is an extremely useful diagnostic technique for both patients and doctors, and its use is expected to become more widespread in the future.
